# Pharmacoinformatic Approach to Explore the Antidote Potential of Phytochemicals on Bungarotoxin from Indian Krait, *Bungarus caeruleus*

**DOI:** 10.1016/j.csbj.2018.10.005

**Published:** 2018-10-31

**Authors:** Barani Kumar Rajendran, M. Xavier Suresh, Shanmuga Priya Bhaskaran, Yarradoddi Harshitha, Uma Gaur, Hang Fai Kwok

**Affiliations:** a Institute of Translational Medicine, Faculty of Health Sciences, University of Macau, Avenida de Universidade, Taipa, Macau; b Department of Physics, Sathyabama Institute of Science and Technology, Deemed to be University, Chennai 600119, India

**Keywords:** Toxins, Bungarotoxin, Molecular docking, Pharmacokinetic profiling, Drug design, Molecular dynamics

## Abstract

Venomous reptiles especially serpents are well known for their adverse effects after accidental conflicts with humans. Upon biting humans these serpents transmit arrays of detrimental toxins with diverse physiological activities that may either lead to minor symptoms such as dermatitis and allergic response or highly severe symptoms such as blood coagulation, disseminated intravascular coagulation, tissue injury, and hemorrhage. Other complications like respiratory arrest and necrosis may also occur. Bungarotoxins are a group of closely related neurotoxic proteins derived from the venom of kraits (*Bungarus caeruleus*) one of the six most poisonous snakes in India whose bite causes respiratory paralysis and mortality without showing any local symptoms. In the current study, by employing various pharmacoinformatic approaches, we have explored the antidote properties of 849 bioactive phytochemicals from 82 medicinal plants which have already shown antidote properties against various venomous toxins. These herbal compounds were taken and pharmacoinformatic approaches such as ADMET, docking and molecular dynamics were employed. The three-dimensional modelling approach provides structural insights on the interaction between bungarotoxin and phytochemicals. *In silico* simulations proved to be an effective analytical tools to investigate the toxin–ligand interaction, correlating with the affinity of binding. By analyzing the results from the present study, we proposed nine bioactive phytochemical compounds which are, 2-dodecanol, 7-hydroxycadalene, indole-3-(4'-oxo)butyric acid, nerolidol-2, trans-nerolidol, eugenol, benzene propanoic acid, 2-methyl-1-undecanol, germacren-4-ol can be used as antidotes for bungarotoxin.

## Graphical abstract

**Figure:**
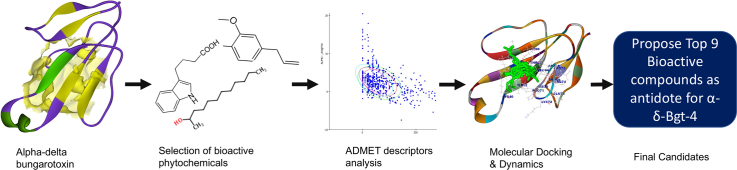

## 1 Introduction

Snake venom (SV) is a mixture of proteins and peptides that perform a myriad of biological functions. Most venomous snakes are found in the Colubridae, Elapidae, and Viperidae families. The major snake bite mortalities are caused by four highly venomous snake species, *Daboia russelii*, *Echis cariatus*, *Naja naja* and *B.caeruleus* or Indian krait which are commonly called as “Indian big four”. Snake bite fatality rates are also considerably increasing due to lack of antivenom, lack of awareness and comparatively slow or poor treatment strategies [[1], [2], [3], [4]].

Snake venoms are comprised of a diverse array of toxins that have a variety of biochemical and pharmacological functions and are classified as hemotoxins, neurotoxins, necrotoxins, cytotoxins, etc. Neurotoxins are the toxins that primarily affect the nervous system by strongly binding with nicotinic acetylcholine receptors (nAchRs). Bungarotoxins are group of closely related neurotoxic proteins which are derived from the venom of kraits. Based on the length of their polypeptide chain, neurotoxins are further classified as, short and long chain neurotoxins. Bungarotoxins are classified as alpha-bungarotoxin, beta-bungarotoxin, kappa-bungarotoxin and gamma bungarotoxin. Alpha-deltabungarotoxin-4 (Alpha-delta-Bgt-4) is one of the potent alpha neurotoxin found in the elapidae family Indian krait usually called *Bungarus caeruleus*. Krait venom is extremely neurotoxic and highly lethal to humans and acts without showing any local symptoms making it the major cause of mortality of snake bite victims [5,6]. The venom of common krait contains the most potent neurotoxins that have both pre-synaptic and post-synaptic neurotoxins and resulting to high binding affinity to muscular and neuronal nicotinic acetylcholine receptor (nAChR), it stimulates muscular paralysis by affecting nerve endings situated near the synaptic cleft of brain cells leading to respiratory paralysis, severe abdominal cramps, leading to death [[7], [8], [9], [10], [11]].

Alpha-neurotoxins, specifically alpha-delta-Bgt-4 binds with high affinity to the acetylcholine receptor and compete with binding of the natural ligand. These toxins play a key role in the isolation and characterization of mammalian nicotinic acetylcholine receptors. Many α-bungarotoxin are antagonists at native GABA(A)receptors [[12], [13], [14]]. The krait bite is treated with antivenom treatment, and it shows several undesirable life threatening side effects such as nausea, urticarial, hypotension, cyanosis, severe systemic anaphylactic reactions that can be a risk to some of the victims [15,16]. The alternative way of treating the snake bite cases are using several plants based bioactive inhibitor compounds, which were used by people in ancient days as folklore medicine to treat the various venomous species bites victims such as, snake, scorpions, etc., and it has shown significant outcome against envenomation [[17], [18], [19]]. Despite the lethality of venom, it has been used widely for many pharmacological applications such as treating several neurological diseases and used for cell cycle arrest, and other venom based anti-cancer therapeutic approaches [[20], [21], [22]].

**Fig. 1:**
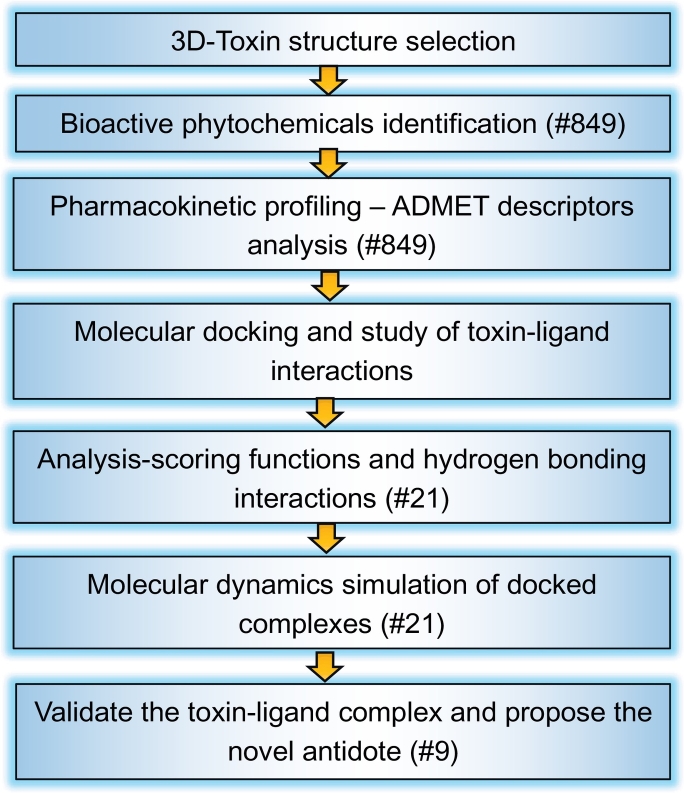
The summarized step-by-step protocol flowchart used in this study: the detailed pipeline procedures from the 3D structure selection to the final inhibitors validation: and the numbers (#) represented the selection of ligand compounds used in each level of analysis.

The main aim and objective of this study is to identify novel antidotes from potential bioactive phytochemicals for snake bites specifically for α-δ-Bgt-4 using pharmacoinformatic approaches including computational three-dimensional structure prediction, high throughput ligand screening, pharmacophore mapping, pharmacokinetic profiling and molecular docking, molecular dynamics (MD) simulation analysis. This in silico study will provide deep insight into molecular properties of selected phytochemicals and their mode of action to neutralize the toxin of *B.caeruleus,*α-δ-bungarotoxin-4. This study may also help to further investigate the possibility of using these bioactive phytochemicals to avoid adverse life threatening side effects caused by antivenom treatment (Fig. 1).

## 2 Materials and Methods

### 2.1 Toxin Structure Preparation, Simulation and Binding Site Prediction

Alpha-delta bungarotoxin is one of the active neurotoxin from *Bungarus caeruleus* and till now there is no experimentally solved 3D structure for this protein. In the absence of experimentally proved structure, the alternative and reliable approach is homology based structure prediction, it gives better outcomes although it fully depends on the template- target protein sequence homology [23,24]. Hence we used homology modelling approach to predict this protein structure and detailed methodology including template protein selection, overall structure quality, etc. were mentioned in our previous study [25]*.* This bungarotoxin binds with high affinity at the neuromuscular junction. The length of the bungarotoxin peptide is 76 amino acids. The homology modelled structure was built using Modeller9v7 by employing regular protocols [[23], [24], [25], [26], [27], [28]] and the structure in PDB format was used for protein preparation including structure stabilization using energy minimization followed by simulation module of Discovery studio with default settings. Simulated structure was further validated with φ, ψ plot and resulting energetically stable structure was used for further molecular docking analysis. Binding site pocket was predicted using binding site prediction tool of Discovery studio and predicted binding site volume was 26.500 Å^3^, which comprises of predicted used for docking analysis.

### 2.2 Collection of Plants and Their Chemical Compounds

Many medicinally engrossed plants species were identified and used for several human ailments in earlier days. Each plant has hundreds of bioactive compounds, and each one has their own biological and medicinal properties. Literature survey enabled the retrieval of 849 herbal compounds isolated from 82 plant species which had already shown significant biological activities. These 849 compounds were screened against the target toxin. Subsequently, the structures of the 849 herbal compounds collected from 82 plants were downloaded from PubChem and Chemspider which were the databases of chemical molecules in ‘sdf’ and ‘mol’ format respectively [[29], [30], [31]]. Some of these compounds were collected in the form of canonical smiles and were saved in ‘smi’ format and these smile notations were opened in discovery studio and optimized. SwissADME tool was used for analyzing ligands with various ADME and drug likeliness evaluation [32]. These herbal compounds were considered as ligands and the target protein here was bungarotoxin.

### 2.3 Pharmacokinetic Profiling-ADMET Studies

This method was used to study the pharmacokinetic properties of the drug molecule such as Absorption, Distribution, Metabolism, Excretion and Toxicity after the oral administration of the drug into the biological system. The ligand molecules were subjected to ADMET studies which helped in filtering the best scored ligands that satisfy conditions such as the ligand absorption level, solubility level, hepatotoxicity level, blood brain barrier level and so on. There were different ranges of values or probabilities for each property studied in the ADMET descriptors. A scatter plot for the ADMET descriptors was obtained and analyzed. The ADMET descriptors offered detailed information of the BBB level (BBB level: 0-Brain-Blood brain ratio greater than 5:1, 1-Brain-blood ratio between 1:1 and 5:1, 2-Brain-blood ratio between 0.3:1 and 1:1, 3-Brain-blood ratio less than 0.3:1), absorption level, solubility level, hepatotoxicity, CYP2D6, PPB level and PSA_2D (polar surface area) values. The BBB level showed the amount of penetration of the drug into the CNS after oral administration of drugs. An optional drug should not penetrate the BBB level as it can cause side effects in the CNS. Thus, the drug compounds with BBB values 2 and 3 were considered optimal for a drug to be administered. The absorption level predicts the human intestinal absorption after oral administration. The absorption level of the drug should be high or medium, i.e., the value should be either 0 or 1 so that the drug is absorbed by the intestines after oral administration for future metabolism. Thus, a drug with absorption level 0 or 1 was considered optimal. The solubility level depicts the drug-likeliness of the given compound. The accepted values were 3 and 4 for an optimal drug. The 0, 1, and 2 values do not satisfy the drug-likeness while drug with value 5 is too soluble which could be too much penetrating causing side effects. A drug compound was toxic or non-toxic based on its effect of causing dose dependent liver injuries. The drug toxicity was predicted based on the hepatotoxicity probability. It becomes toxic at a probability >0.5 and nontoxic if it was less than 0.5 depending upon the doses. The PPB level was also known as the Plasma Protein Binding level of drugs which estimated the binding of the drug to the plasma membrane based on the atom based logarithmic partition coefficient. The values are 0 – binding <90%, 1 – binding >=90%, 2 – binding >95%.

### 2.4 Molecular Docking

This method predicted the preferred orientation of one molecule to a second molecule when bound to each other to form a stable complex. Discovery studio is a single, powerful, easy-to-use, graphical interface for drug design and protein modelling research. It supports a variety of algorithms for ligand designing, protein-ligand docking, simulation studies and so on. Active site prediction was carried out to identify the active site residues of the protein. Receptor-ligand interactions were studied using discovery studio with appropriate algorithm. The ligand-fit algorithm was employed for performing the receptor-ligand docking for the selected ligand molecules with the target at the identified binding site of the protein. The docked molecules were viewed for hydrogen bond interactions between the ligand atoms and the amino acids residues of the receptor molecule. The distance between the bonds was calculated and estimated for studying the favorable interactions between the ligand and the receptor molecule. Various interactions such as the alkyl bonds, pi bonds, and the respective distances were calculated for the receptor-ligand complex using Discovery studio 2.0.

### 2.5 Study of Receptor-ligand Interactions and Simulation of Docked Complex

The hydrogen bond interactions between the ligand and receptor molecule retained after docking was studied and analyzed in the discovery studio. The hydrogen atoms, interacting atoms, the amino acid residues of the receptor molecule were labelled. The hydrogen bond interactions were monitored using the software and the bond length is calculated. The distance between the interacting amino acid residues and the ligand molecule atoms were calculated. Docked toxin-ligand complexes were simulated by using simulation module of Discovery studio software. The standard dynamics cascade protocol was used for 50 nano second (ns) simulation for each complex with default parameters. Prior to simulation the molecule was minimized using Steepest descent and conjugate gradient algorithms and subjected to heating and equilibration with NVT ensemble. The temperature were set to 300K with 50K increases and further MD trajectories were analyzed for studying stability of binding and inter molecular interactions. The root mean square deviation and root mean square fluctuations of the residues were also calculated.

## 3 Results

### 3.1 Selection of Target Protein

**Fig. 2:**
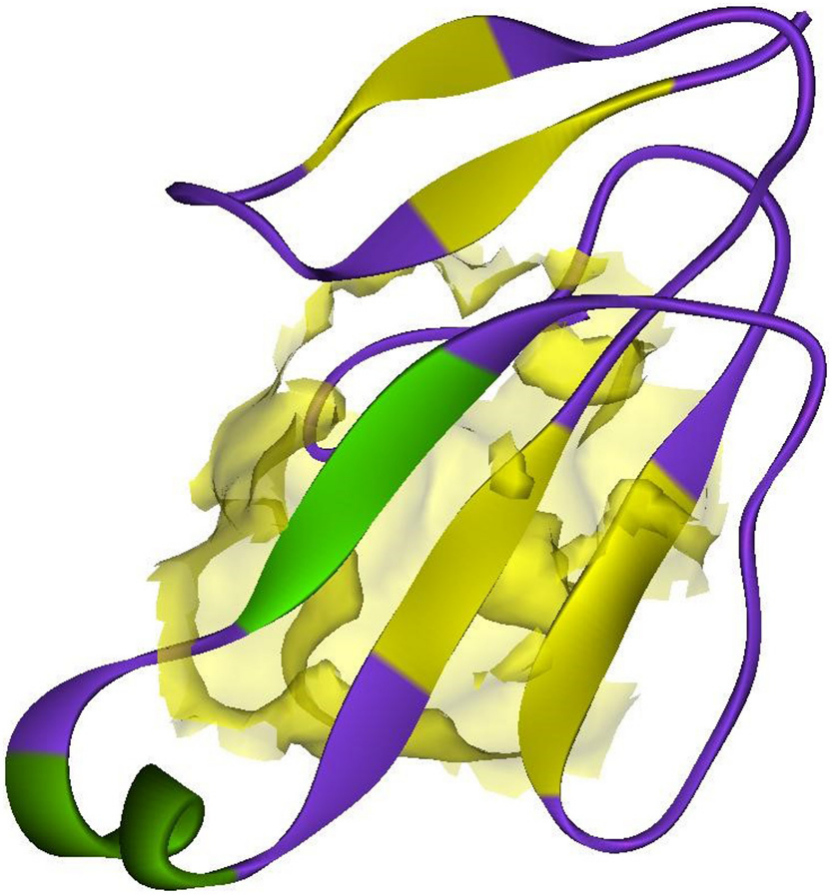
Three dimensional structure of alpha-delta-bungarotoxin-4 and its binding site were highlighted with surface view.

Alpha-bungarotoxin and kappa-bungarotoxin belongs to three-finger toxin family [14]. These three-finger toxins are a group of low molecular weight toxins which are small proteins. These small proteins are non-enzymatic polypeptides with molecular weight of less than 10KDa, and having length of 60-74 amino acid residues. They have 8-10 cysteine residues which form four or five disulfide bridges, of which four of the disulfide bridges are highly conserved among these kinds of toxin family proteins. Toxin members of this family have similar protein structures, three beta stranded loops which extends from a central core and is made up of four highly conserved disulfide bonds. The alpha-delta-bungarotoxin-4 consists of 76 amino acids with the molecular weight of 8292.71 KDa (https://www.uniprot.org/uniprot/D2N116) [33,34]. The sequence information of the α-δ-Bgt-4 had been obtained from the SwissProt database and the three-dimensional structure was built using computational homology modelling approach using Modeller9v7 [25]. The modelled structure was validated using Structure Analysis and Verification Server (SAVS) [35]. The Ramachandran plot showed the phi, psi angles of almost all the residues are in favorable and allowed regions. The overall architecture followed the three-finger fold as observed for other short chain neurotoxins. Furthermore, the pharmacophore analysis of the predicted binding sites revealed that the pockets appeared prevalently hydrophobic, however, with H-bond acceptors and donors. The 3D structure of the bungarotoxin was viewed in the Discovery Studio software and showed in Fig. 2.

### 3.2 Selection of Bioactive Phytochemicals From the Medicinal Plants

The HERBMED(http://www.herbmed.org/) and Dr. Duke’s database for medicinal plants were used for identifying herbal compounds. From these databases and further rigorous literature study, 849 bioactive phytochemical constituents were identified from 82 medicinal plant species. The structures of the 849 herbal compounds were retrieved from PubChem database. The total numbers of compounds from the individual plant species were listed in Table 1 and the detailed list of bioactive compounds and their PubChem Identifiers and canonical SMILES notations were given in the Supplementary Table 1.

**Table 1:** List of plants and their bioactive phytochemical compounds identified and used in this study

Sl.no.	Plant name	Number of compounds
1.	Andrographisechdes nees	23
2.	Achyranthus aspera L	17
3.	Aristolochia indica L	15
4.	Boerhavia diffusa L	5
5.	Leucas aspera	13
6.	Cissus repens lamk	2
7.	Moringa oleifera lam	16
8.	Ficus benghalensis L	12
9.	Drymaria cordata wild	12
10.	Andrographis stenophylla	2
11.	Tamarindus indica. L	10
12.	Acorus calamus	20
13.	Andrographis paniculata	4
14.	Calotropis gigantean	3
15.	Emblica officinalis	11
16.	Euphorbia neriifolia	15
17.	Gymnema sylvestre	15
18.	Kalanchoe pinnata	18
19.	Mimosa pudica	6
20.	Rauvolfia serpentine	14
21.	Tinospora cordifolia	13
22.	Vitex negundo	20
23.	Withania somnifera	8
24.	Aristolochia odoratissima	65
25.	Cissus assamica	7
26.	Echinacea angustifolia	9
27.	Guiera senegalensis	6
28.	Hemidesmus indicus	4
29.	Parkia biglobosa	10
30.	Securidaca longipedunculata	9
31.	Trianosperma tayuya	8
32.	Thea sinesis	18
33.	Alangium salvifolium	3
34.	Cissampelos	6
35.	Barleria prionitis	10
36.	Helicteres isora L	9
37.	Holarrhena pubescens	5
38.	Lantana indica Roxb	6
39.	Ammannia baccifera L	7
40.	Andrographis serpyllifolia	15
41.	Anogeissus latifolia	19
42.	Atalantia racemosa	10
43.	Bacopa monnieri	11
44.	Bixa orellana	4
45.	Calycopteris floribunda	6
46.	Carmona retusa	12
47.	Cassine glauca	3
48.	Ceiba pentandra	23
49.	Corallocarpus epigaeus	11
50.	Derris scandens	11
51.	Desmodium motorium	7
52.	Dichrocephala integrifolia	8
53.	Ehretia canarensis	4
54.	Hedyotis puberula	10
55.	Hoppea dichotoma	30
56.	Hugonia mystax	25
57.	Justicia tranquebariensis	11
58.	Lantana indica	4
59.	Luffa cylindrical	8
60.	Murraya paniculata	17
61.	Naringi crenulata	17
62.	Stereospermum colais	4
63.	Ochna obtusata	1
64.	Opilia amentacea	4
65.	Polyalthia korinti	6
66.	Tylophora indica	7
67.	Vicoa indica	5
68.	Wattakaka volubilis	5
69.	Albizia lebbeck	4
70.	Annona squamosa	19
71.	Aristolochia bracteolate	10
72.	Corallocarpus epigaeus hook	15
73.	Datura metel	7
74.	Dichrostachys cinerea	2
75.	Diplocyclos palmatus	4
76.	Enicostema axillare	14
77.	Evolvulus alsinoides	5
78.	Helicteres isora	3
79.	Hygrophila auriculata	1
80.	Justicia simplex	9
81.	Madhuca indica	6
82.	Plumbago zeylanica	8

### 3.3 Pharmacokinetic Profiling –ADMET Analysis

**Fig. 3:**
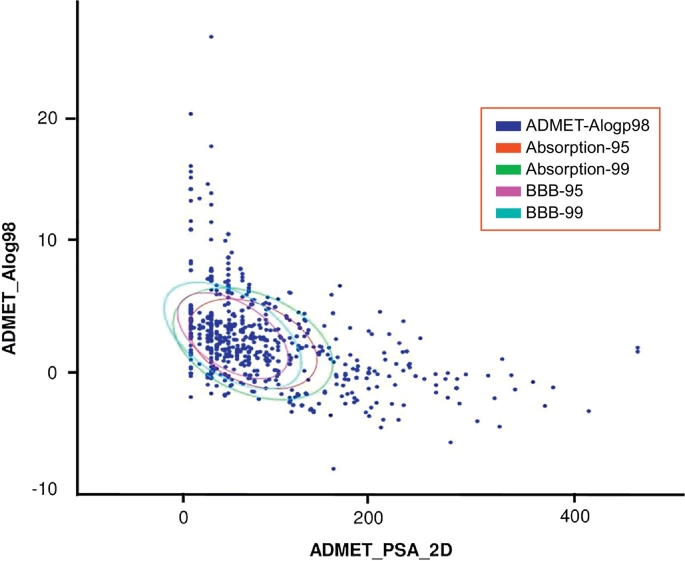
ADMET descriptors of all selected ligand molecules and their Alogp98, absorption and blood brain barrier penetration (BBB) confidence levels (95 and 99) are shown in different color circles.

The drug likeliness, ADME and pharmacokinetic properties of selected bioactive ligand molecules were checked for their drug like features using SwissADME tool (http://www.swissadme.ch/index.php). In this analysis, we used twenty different ADME and drug likeliness parameters to evaluate the ligands (Supplementary Table 2). Further validation of the selected bioactive compounds was carried out using ADMET (Absorption, Distribution, Metabolism, Excretion and Toxicity) descriptors module of Discovery Studio and the results (point plots) descriptors analysis were carried out for all the 849 compounds and the detailed results were illustrated in Fig. 3. Out of 849 compounds, 34 compounds satisfied and passed all levels of the ADMET descriptors and were used for further analysis. The detailed ADMET descriptors analysis scores are given in Table 2.

**Table 2:** ADMET descriptor analysis of high confidence bioactive compounds with Polar surface area (PSA).

PubChem ID	BBB^a^	Absorption	Solubility	Hepatotoxicity	CYP2D6	PPB	PSA_2D
10256	2	0	3	0	0	2	32.35
5284507	0	0	3	0	0	1	20.81
11005	0	0	2	0	0	2	38.11
4113470	0	0	2	0	0	2	34.60
535214	0	0	3	0	0	1	20.81
73337	3	0	3	0	0	2	59.49
6492375	0	0	3	0	0	1	20.81
3314	1	0	3	0	0	2	29.74
730037	3	0	3	0	0	2	70.47
445070	0	0	3	0	0	1	20.81
91522	1	0	3	0	0	2	53.02
8888	0	0	3	0	0	1	20.81
73187	1	0	3	0	0	2	26.23
75187	1	0	3	0	0	2	26.23
608115	0	0	2	0	0	2	20.81
637563	1	0	3	0	0	2	18.93
8193	0	0	3	0	0	1	20.81
117464	1	0	3	0	0	2	31.11
66341	0	0	3	0	0	0	20.81
73117356	2	0	3	0	0	2	53.50
73117	2	0	3	0	0	2	53.50

a BBB level: 0-Brain-Blood brain ratio greater than 5:1, 1-Brain-blood ratio between 1:1 and 5:1, 2- Brain-blood ratio between 0.3:1 and 1:1, 3-Brain-blood ratio less than 0.3:1; Solubility level-3: good soluble (range from -4.0 <log(Sw)<-2.0); Absorption level: 0 stands for good absorption T2_2D<6.1261(inside 95% confidence level), Hepatotoxicity: 0- Nontoxic (Hepatotoxicity probability is <0.5; CYP2D6:0-Non-inhibitor, unlikely to inhibit CYP2D6 enzyme. PPB- Plasma Protein Binding:0-binding is <90% and AlogP98<4.0, 1-binding is ≥90% and AlogP98≥4.0, 2-binding is ≥95% and Alogp98≥5.0; PSD: Polar Surface Area, metric for the optimization of a drug’s ability to infuse cells.

### 3.4 Molecular Docking and Analysis of Toxin-ligand Interactions

**Fig. 4:**
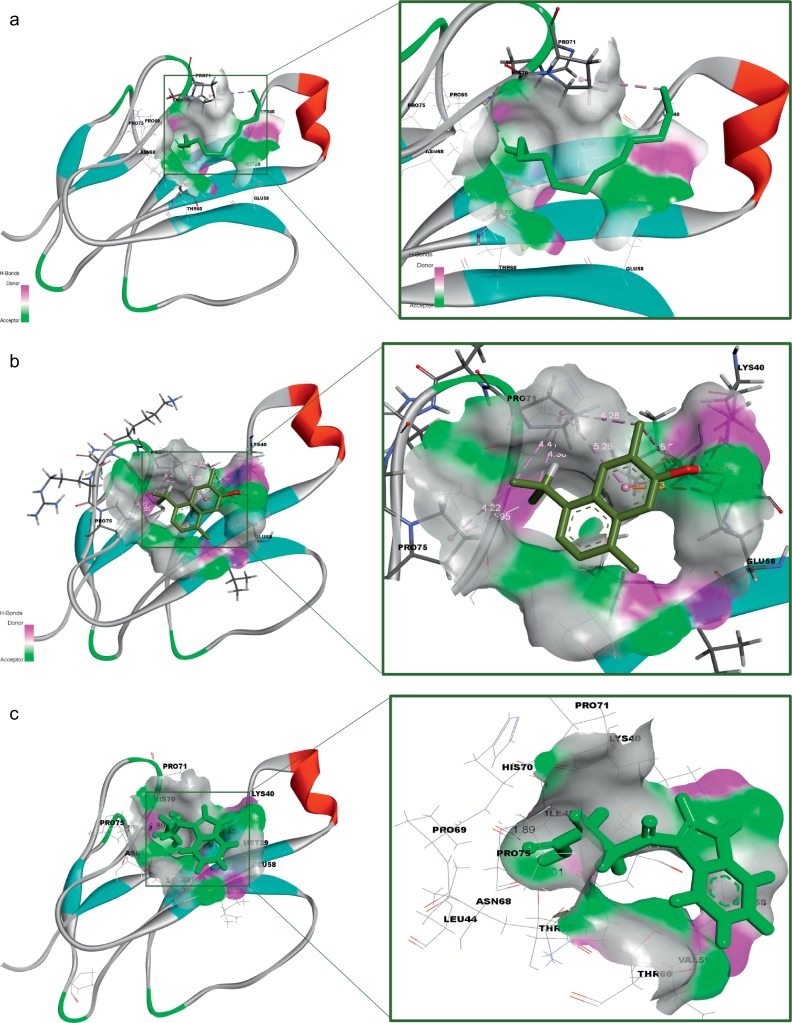

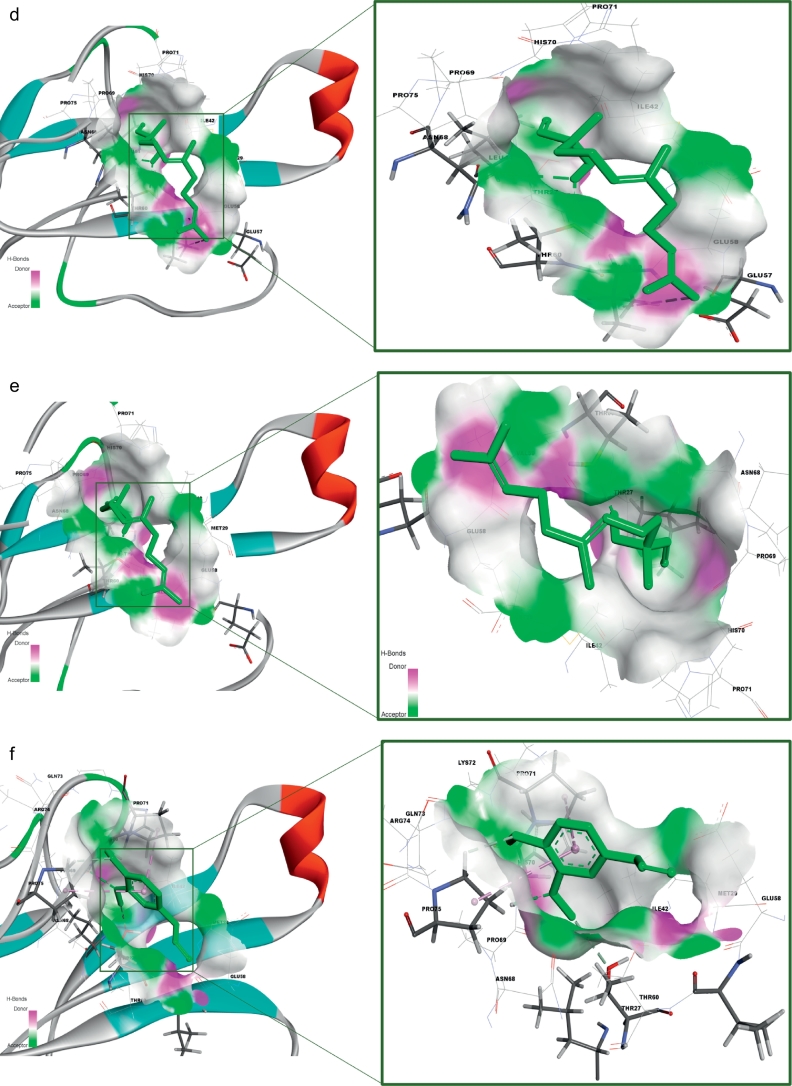

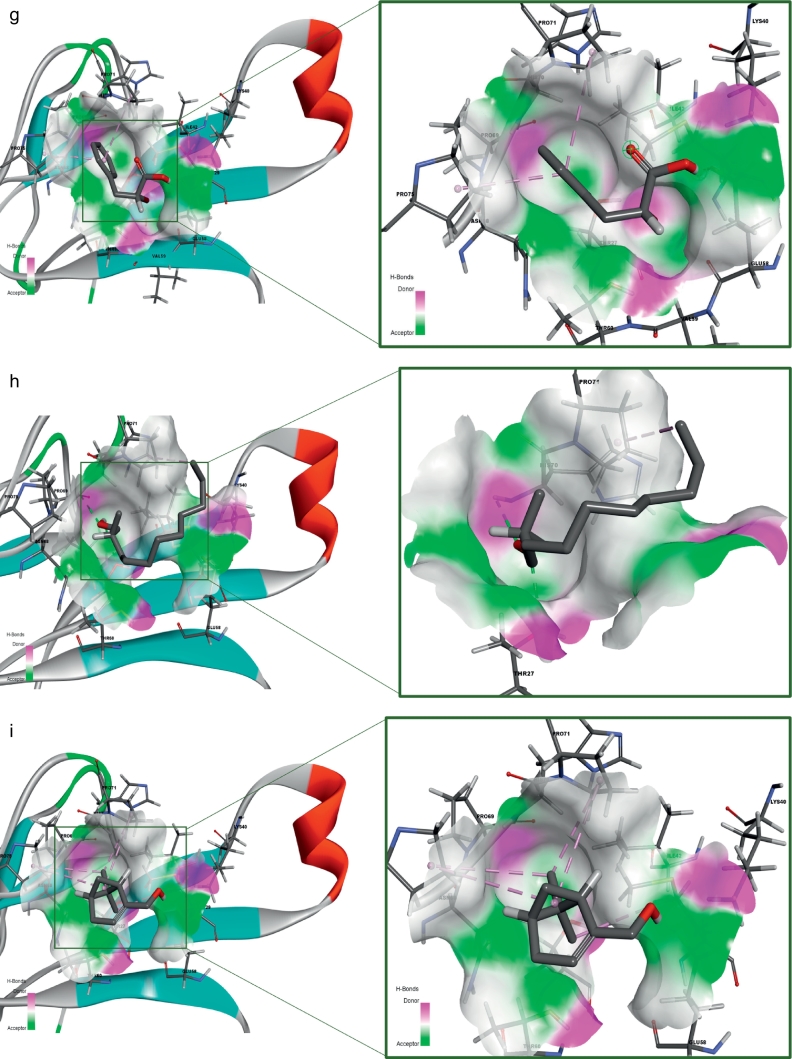

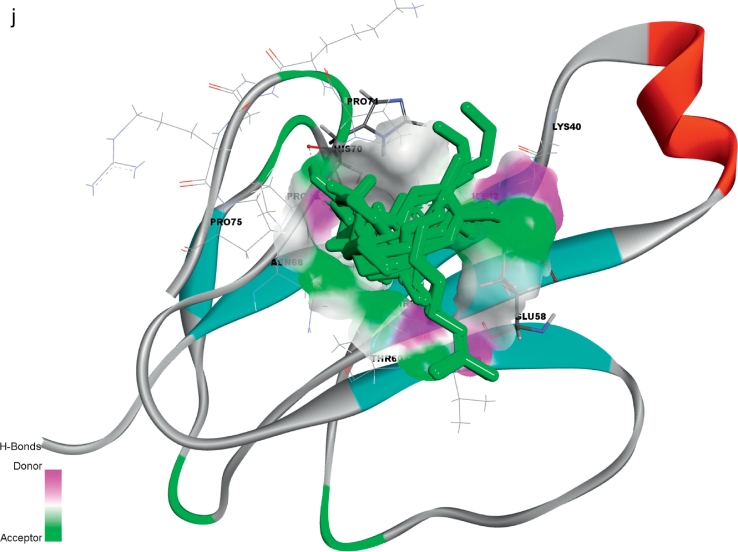
Receptor (toxin)–ligand interactions were assessed using molecular docking of α-δ-Bgt-4 with corresponding ligand molecules. Left side of each panel was overall toxin-ligand complex with secondary structure view and right side of the panel was atom level interactions of (A) 2- Dodecanol, (B) 7- Hydroxycadalene, (C) Indole-3-(4'-oxo)butyric acid, (D) Nerolidol-2, (E) Trans-nerolidol, (F) Eugenol, (G) Benzene propanoic acid, (H) 2-methyl-1-undecanol, (I) Germacren-4-ol, (J) overall all interactions of all selected ligands with α-δ-Bgt-4 toxin.

The receptor (α-δ-Bgt-4 toxin) and selected ligand molecules were docked using the Ligand-Fit algorithm of Discovery Studio 2.0. The detailed amino acid interaction profiles, binding energy of the compounds and other scoring functions were shown in Table 3. The inter-molecular interaction results were illustrated in Fig. 4.

**Table 3:** Molecular docking interaction results α-δ-Bgt-4 toxin with selected bioactive compounds.

Compound PubChem ID	Rotational bonds	Ligand internal energy (Kcal/mol)	Ligand score1	Ligand score2	PLP 1	PLP 2	Jain	PMF	Dock score
8193	10	−2.649	2.36	3.93	40.67	39.27	–1.94	29.11	30.55
608115	2	−2.419	0.69	1.81	17.13	25.29	−0.84	24.83	30.157
730037	4	−1.361	2.23	2.9	20.07	19.93	−1.59	25.8	29.554
5284507	7	−2.347	1.71	3.62	41.19	47.85	−1.66	8.81	28.361
3314	3	−1.744	3.2	3.85	39.45	37.56	−0.05	10.5	27.813
117464	4	2.986	1.25	2.65	26.41	28.18	−0.38	11.57	27.317
6429375	2	0.102	1.48	2.88	19.5	21.12	−1.11	21.14	26.867
66341	9	−2.997	2.05	3.46	27.16	32.61	−0.92	18.68	26.689
11005	12	−3.07	1.51	3.3	35.78	37.22	−0.82	19.44	26.041
535214	7	−4.309	1.27	2.91	32.28	38.8	−1.94	5.49	24.554
637563	2	−1.529	1.15	3.11	30.66	29.89	−1.21	16.64	23.560
75187	3	−1.603	1.25	3.25	26.42	25.02	−1.08	32.89	23.153
356	3	−1.524	0.64	3.11	28.08	29.35	−1.54	10.29	22.837
445070	8	−3.418	1.96	3.37	36.1	38.31	−0.82	24.87	22.749
528155	2	−1.14	0.66	3.12	28.47	29.81	−1.37	11.87	22.455
14896	2	−1.174	0.64	3.09	29.2	30.05	−1.37	15.33	22.114
546270	2	−1.114	0.59	3.02	29.17	29.86	−1.28	15.31	21.014
8888	7	−1.994	0.5	2.78	35.26	39.22	−2.01	2.07	20.174
22311	0	−1.05	0.56	2.98	27.03	31.07	−0.65	8.5	19.399
8058	1	−0.563	0.49	2.86	22.37	23.77	−1.09	1.84	18.510
91522	0	−2.277	0.31	2.47	37.49	41.04	1.39	−15.3	13.640

Dock score was summation of few scoring functions such as PLP1, PLP2, Ligand scores, Jain and PMF. Hence, there was no unit for Dock score and greater Dock score indicated better binding. Foot note was added to Table 3 for each scoring function. PLP: Piecewise Linear Potential was simple and fast docking function that had been shown to correlates well with protein-ligand binding affinities. Higher PLP scores showed stronger receptor-ligand binding. PLP1 calculates H-bond donor only, H-bond acceptor only, both H-bond donor and acceptor and non-polar atoms. PLP2 calculated same as PLP1, in addition, an atomic radius was assigned to each atom except H. Ligand Score1 and 2: was also a fast and simple scoring function for predicting receptor-ligand binding affinities. It used vdW, C+pol descriptors and total polar surface area of receptor and ligand molecules. Ligand score 2 was also similar like Lig.score1, In addition, it used buried polar surface area of the receptor and the ligand molecules. Jain was an empirical scoring function, it calculated binding affinities of docked molecules using five parameters such as lipophilic, polar attractive and repulsive interactions, solvation of the receptor and ligand and an entropy of the ligand. PMF (Potentials of Mean Force): This score was calculated by summing pairwise interaction terms over all interatomic pairs of the complex. Dock score was calculated based on force field approximation by using the formula (Dock score (force field)) = (ligand/receptor interaction energy + ligand internal energy) and other on the PLP (Dock score(PLP)=-(PLP potential)).

**Fig. 5:**
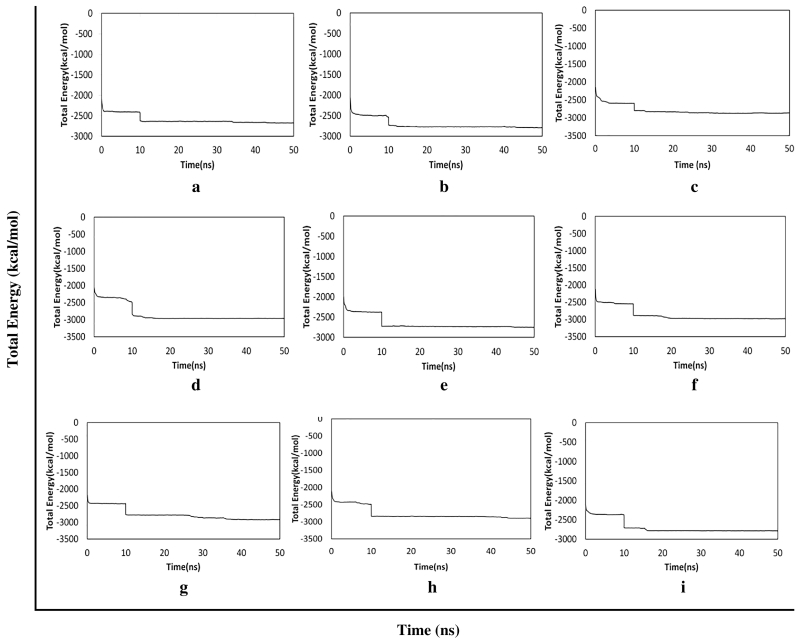
Graphical representation of the total energy profile of α-δ-Bgt-4 complex with (a)2-dodecanol, (b)7-hydroxycadalene, (c)Indole-3-(4'-oxo) butyric acid, (d)Nerolidol-2, (e)Trans-nerolidol, (f)Eugenol, (g)Benzene propanoic acid, (h)2-methyl-1-undecanol, (i)Germacren-4-ol.

Table 3, demonstrated the results for docking performed by giving ten poses for the receptor-ligand interactions as input but in the table only top score was mentioned. From the 849 compounds, only 34 compounds docking poses were successfully generated with 335 poses. The ligand score, PMF, PLP, Jain, dock score were the five parameters which were used to analyze the docking results. Higher dock score indicated the better interaction which was calculated by the sum of five interaction terms including lipophilic interactions, polar attractive interactions, polar repulsive interactions, solvation of the protein and ligand and an entropy term for the ligand. Based on the docking results obtained top ten compounds were taken and subjected to further analysis of hydrogen bond interaction studies that helped determine the stability of the molecular complex. The interactions were studied and analyzed for all the ten compounds. List of the compounds were listed in the Table 4 and the total energy of their docked complexes were depicted in Fig. 5.

**Table 4:** List of the best docked bioactive inhibitor compounds and their amino acid interactions.

Name of the plant	Name of the compound	Dock score	H-bond interaction	Interacting residue	H-Bond distance (Å)
*Hugonia mystax*	2-dodecanol	30.55	1	THR27	2.37
*Ceiba pentandra*	7-hydroxycadalene	30.157	2	GLU58	2.21
				GLU58	1.12
*Withania somnifera*	Indole-3-(4'-oxo) butyric acid	29.554	3	THR27	2.01
				PRO69	2.50
				HIS70	1.89
*Leucas aspera*	Nerolidol-2	28.361	1	THR60	2.30
*Murraya paniculata*	Trans-nerolidol	28.343	1	THR60	2.08
*Annona squamosa*	Eugenol	27.813	2	HIS70	2.47
				HIS70	1.96
*Hugonia mystax*	Benzene propanoic acid	27.317	2	GLU58	2.33
				GLU58	1.22
*Hugonia mystax*	2-methyl-1-undecanol	26.689	1	HIS70	2.32
*Vitex negundo*	Germacren-4-ol	26.867	1	GLU58	1.34

### 3.5 Molecular Dynamics Simulations

Further the top scored toxin-bioactive plant compound complexes were subjected to a MD simulation for a period of 50ns. Initially the toxin-ligand complex structures were prepared and typed with CHARMm forcefield and subjected to energy minimization using Steepest Descent (SD) and Conjugate Gradient (CG) algorithms to remove the modelling artifacts. Further, solvation, heating, equilibration and production steps were followed for a period of 50 nano second (ns) prior NVT ensemble used and temperature was set to 300 kelvin (k). Production results were processed and total of 450ns molecular dynamics trajectories of each toxin-ligand complex were further analyzed. The result of simulation total energy profile all selected complexes are illustrated in Fig. 5.

From the above graph, obviously the initial and final potential energies were in the range -2600 Kcal/mol to -3000 Kcal/mol. The stable energy levels were achieved around -3000 kcal/mol. The trajectory analysis revealed the total energy profiles were established around 10 to 12ns duration. From this analysis we also observed the stability of hydrogen bonding interactions. The potential energy profile of the simulated bungarotoxin-bioactive ligand complexes along with other energy terms are given in Table 5.

**Table 5:** The various energy profiles of all simulated toxin-ligand complexes.

Compound	Force field	Temp (K)	Initial potential energy	Total energy	Final potential energy	Kinetic energy	VdW energy	Electrostatic energy
			(kcal/mol)					
2-dodecanol	CHARMm Force field	304.18	-3093.154	-2678.914	-3529.786	850.871	-366.464	-4697.598
7-hydroxycadalene		300.24	-3069.620	-2795.537	-3628.304	832.767	-369.599	-4772.393
Indole-3-(4'-oxo)butyric acid		301.08	-3173.005	-2862.668	-3692.061	829.393	-389.085	-4797.730
Nerolidol-2		303.23	-3724.008	-2968.220	-3845.283	877.062	-413.722	-4784.957
Trans-nerolidol		301.24	-3593.597	-2761.020	-3632.325	871.304	-345.022	-4624.080
Eugenol		295.98	-3743.698	-2983.106	-3827.423	844.316	-378.310	-4772.548
Benzene propanoic acid		305.47	-3649.963	-2915.503	-3785.070	869.566	-368.337	-4742.657
2-methyl-1-undecanol		300.46	-3704.977	-2905.814	-3772.166	866.351	-412.778	-4734.256
Germacren-4-ol		306.49	-3563.158	-2796.585	-3672.413	875.828	-373.087	-4635.080

**Fig. 6:**
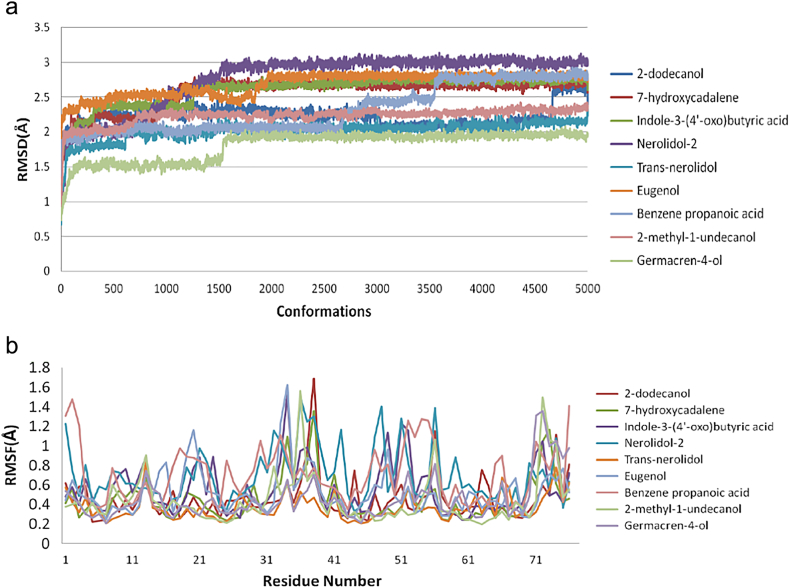
(a) RMSD value for all top nine toxin-ligand complexes are shown and overall structural level RMSD deviations were recorded for 5000 conformations; (b) Similarly RMSF were calculated for individual amino acid residue level and almost 90% amino acids RMSF were less than 1.6 Å.

Further, root mean square deviation (RMSD) and root mean square fluctuation (RMSF) were calculated for all simulated complexes using molecular dynamics trajectory analysis tool and detailed plots were shown in Fig. 6.

## 4 Discussion

Snake venom components are studied for various reasons including their applications in biomedicine and these toxins may be used as a tool for targeted therapy for many diseases. The α-δ-Bgt-4 is a potent neurotoxin produced by highly venomous species from the Elapidae family of serpents, causes death without showing any local symptoms becoming the main cause for death of the bite victims [[36], [37], [38]]. The mechanism of resistance to snake toxins have also been studied through molecular modeling and structural analysis [[39], [40], [41]]. It has been also reported that a 13-mer peptide binds alpha-bungarotoxin with high affinity and neutralizes its toxicity [[42], [43], [44]]. The α-δ-Bgt-4 toxin from *Bungarus caeruleus* is a close homolog of bungarotoxin from *Bungarus multicinctus*, both share a sequence similarity of 92%. By homology it was inferred that alpha-delta-bungarotoxin mainly targets neuronal acetylcholine receptors and specifically binds with high affinity to muscular and neuronal nicotinic acetylcholine receptor (nAChR) and inhibits acetylcholine from binding to the receptor, thereby impairing neuromuscular and neuronal transmission leading to mortality. In this work, due to absence of experimentally proved structure, the three dimensional structure of alpha-delta-bungarotoxin-4 obtained using homology modeling approach. The reliability of the homology derived model was assessed by Ramachandran plot and by checking the internal energy of the protein molecule and found to be reasonable.

A total of 849 plant based bioactive phytochemical compounds were chosen from 82 plants and analyzed for their potential use as antidotes for bungarotoxin through modern pharmacoinformatic techniques. For example, in *Hugonia mystax,* 62 chemical constituents are identified and these chemical compounds have antimicrobial, antibacterial and antifungal activities [45]. In *Leucas aspera* one tenth of the maximum tested dose of the extract of compounds were selected for the evaluation of the hepatotoxic activity [46]. Several of them are tested on animal and human models and the plants are relatively safe for herbal oral medication [[47], [48], [49]]. Scoring and analysis of hydrogen bonding and hydrophobic interactions enable to choose the best compounds, however preference over other toxins would be found by doing a detailed analysis. These plant-based compounds are taken and molecular interaction studies are performed on to them utilizing pharmacoinformatic applications like ADMET, molecular docking and molecular dynamics simulation to enhance our understanding at molecular and atomic level. The ADMET properties of compounds play a crucial role in the drug discovery process as these are largely responsible for around 60% failure of drugs during various clinical phases. Several of the selected herbal compounds did not pass the ADMET screening.

Further toxin-ligand interactions (Ligand-Fit) and molecular dynamics results of the 849 bioactive compounds with bungarotoxin were analyzed based on the dock score and the stability of interactions between the toxin and ligand complex during 50ns molecular dynamics simulations correlating with the binding affinity. For example, the potential energy and kinetic energy profiles of Indole-3-(4'-oxo)butyric acid –toxin complex was initially stabilized by H-bond interactions with Glu58 residue. We observed a hydrophobic interaction with the residues Thr27 and Thr60 whose methyl groups form a hydrophobic pocket which stabilizes the complex. While, Pro69 and Asn68 side chains were at water mediated H- bonding interactions which gives a small change in the position of the ligand binding with the receptor. However, the overall stability of the complex was unchanged. The compounds with better score and favourable interactions were 2-dodecanol, 7-hydroxycadalene and indole-3-(4'-oxo)butyric acid and Nerolidol-2. Nevertheless, 10ns simulations are enough to ascertain the stability of the complex interactions, further simulations were extended to 50ns for the toxin-ligand complexes in order to check the overall stability of the complex. The stable energy levels were achieved around -2800 to -3000kcal/mol and the trajectory analysis revealed that the total energy profile was stabilized around 10 to 12ns and . H-bonding interactions were also found stable.

The structural insights of all the conformations from the computed trajectories revealed that, apart from H-bonding interactions, hydrophobic-hydrophobic interactions were other important driving force offered by the partial hydrophobic environment close to the aperture of binding pocket. These nonbonding interactions help the molecules getting deeply buried inside the binding site. Similar effect was observed in the other complexes nonetheless with slight variations. However, the formation of the additional H- bond was responsible of the higher score recorded for indole-3-(4'-oxo)butyric acid with toxin and contribute to the binding affinity as well. The inspection of RMSD and RMSF plots reveals that, the deviations were in the accepted levels (~1.5 Å) and residue fluctuations were at an average 0.5Å. The fluctuation observed in the RMSF plot is mainly in the region consisting of residues from 32 to 39, which are the middle finger region and 69-71 the terminal loop region. There is no significant conformational changes were observed in the overall structural stability upon ligand binding, however the change in conformation of the side chains of the selected residues away from the binding site may significantly alter the binding affinity of the toxin to the receptor which may eventually alter protein-protein interaction site. From this analysis, nine compounds are proposed as potential inhibitors against α-δ-Bgt-4 which are, 2-dodecanol, 7-hydroxycadalene, Indole-3-(4'-oxo)butyric acid,nerolidol-2, trans-nerolidol, eugenol, benzene propanoic acid, 2-methyl-1-undecanol, germacren-4-ol. They have the preference over the other compounds since some of these already been used in ancient medicine as potential antidotes for bungarotoxin from *B. caeruleus*.

## 5 Conclusions

Venomous animals are famed for their adverse effects after accidental interactions with humans. In this study, several pharmacologically active plants and their phytochemical compounds are collected which are used as antidotes for bungarotoxins, a group of closely related neurotoxic proteins derived from the venom of kraits. The homology modeled three dimensional structure of alpha-delta-bungarotoxin-4 was used in this study to screen several of the plant based compounds. Further by employing computational studies like ADMET, molecular docking and molecular dynamics simulations helps us to study the molecular interaction of these pharmacologically active phytochemical compounds from medicinal plants with alpha-delta-bungarotoxin. In conclusion, the pharmacoinformatic studies proved to be effective analytical tools to investigate the toxin–ligand interactions which in turn helps to uncover the antidote potential of the herbal compounds. Specifically, this study provided structural insights on the bungarotoxin–bioactive phytochemical compound interaction of nine compounds which are, 2-dodecanol, 7-hydroxycadalene, indole-3-(4'-oxo)butyric acid, nerolidol-2, trans-nerolidol, eugenol, benzene propanoic acid, 2-methyl-1-undecanol, germacren-4-ol and these plant based compounds may be suggested as antidotes against bungarotoxin.

## Conflict of Interest Statement

The authors declare that we have no conflict of interest.

## Appendix A Supplementary data

Supplementary material 1 Image 1

Supplementary material 2 Image 2
